# Acute arterial occlusion following inadvertent infusion of ferric hydroxide sacarate (Noripurum®) into the radial artery: case report

**DOI:** 10.1590/1677-5449.202500552

**Published:** 2026-03-23

**Authors:** Daniel César Magalhães Fernandes, Daniel Rassi Gusmao, Felipe Mendonça Oliveira e Souza, Juliano Ricardo Santana dos Santos, Beatriz Franco Fidalgo

**Affiliations:** 1 Hospital Geral de Goiânia – HGG, Goiânia, GO, Brasil.; 2 Instituto de Angiologia de Goiânia – IAG, Goiânia, GO, Brasil.; 3 Universidade Federal de Goiás – UFG, Hospital das Clínicas, Goiânia, GO, Brasil.; 4 Einstein, Hospital Municipal de Aparecida – HMAP, Goiânia, GO, Brasil.

**Keywords:** arterial occlusion, oxidative stress, vasculitis, case report

## Abstract

Iron overload, whether acute or chronic, has been implicated in the pathogenesis of ischemic cardiovascular diseases. However, its effects on peripheral vascular function and thrombotic responses to endothelial injury remain incompletely elucidated. We present a case of inadvertent intra-arterial infusion of ferric hydroxide saccharate (Noripurum®), resulting in severe acute arterial occlusion, most likely secondary to diffuse endothelial injury. Despite prompt surgical intervention and adjunctive pharmacological therapy, the clinical outcome was unfavorable. Given the lack of similar reports in major scientific databases and the involvement of complex, yet poorly characterized, interactions between iron overload and endothelial dysfunction, this case contributes valuable insights into the vascular implications of parenteral iron administration.

## INTRODUCTION

While iron is essential for many physiological processes, in excess it can trigger damage to tissues because of production of reactive oxygen species (ROS) generated by complex reactions.^1^ Secondary iron overload can be caused by frequent blood transfusions or parenteral infusions of substances.^2^ While controversies remain, several bodies of evidence suggest that excess iron can predispose to development of vascular diseases. Elevated levels of iron stores in the body were associated with an increased risk of myocardial infarction in a large cohort of men from Finland.^3^

*In vitro* studies have demonstrated that iron acutely induces platelet aggregation,^4^ whereas iron chelation can inhibit expression of tissue factor.^5^ However, the effects of chronic and acute iron overload on endothelium and the coagulation cascade are still poorly understood.

Iron can also exert direct effects on vascular structures, because local ROS production reduces bioavailable nitric oxide (NO) levels, compromising vascular wall relaxation, predisposing to platelet adhesion and aggregation as a consequence.^6^ There is therefore a relationship between endothelial dysfunction and thrombosis, both potentially mediated by oxidative stress.^7^

Recent studies have shown that after intravenous iron infusions are administered for supplementation, there is a greater than five times increase in levels of non-transferrin-bound iron. While the clinical significance of this increase has not been fully elucidated, *in vitro* evidence indicates that iron in this form can act as a catalyzer of generation of toxic ROS.^8^ The same studies emphasize the importance of the endothelium — in particular of endothelial NO — as a primary mechanism of defense against development of atherosclerotic lesions.^9,10^

In a murine model of acute iron overload in conjunction with hemolytic reaction, Asperti and Vinchi^11^ demonstrated iron- and oxidative stress-mediated endothelial injury that was partially reversed using carbon-monoxide-releasing molecules.^11^

In view of the changes to vascular endothelium induced by iron, and with the objective of improving understanding of this interaction and its possible complications, we present the following case report.

## CASE REPORT

The patient was a 41-year-old female who sought care at an emergency room during the postoperative period after plastic surgery, complaining of weakness, with no other associated conditions. She was given two units of packed red blood cells. The following day, she returned to the urgent care center with similar symptoms and was prescribed intravenous infusion of ferric hydroxide saccharate (Noripurum®).

**Figure 1:**
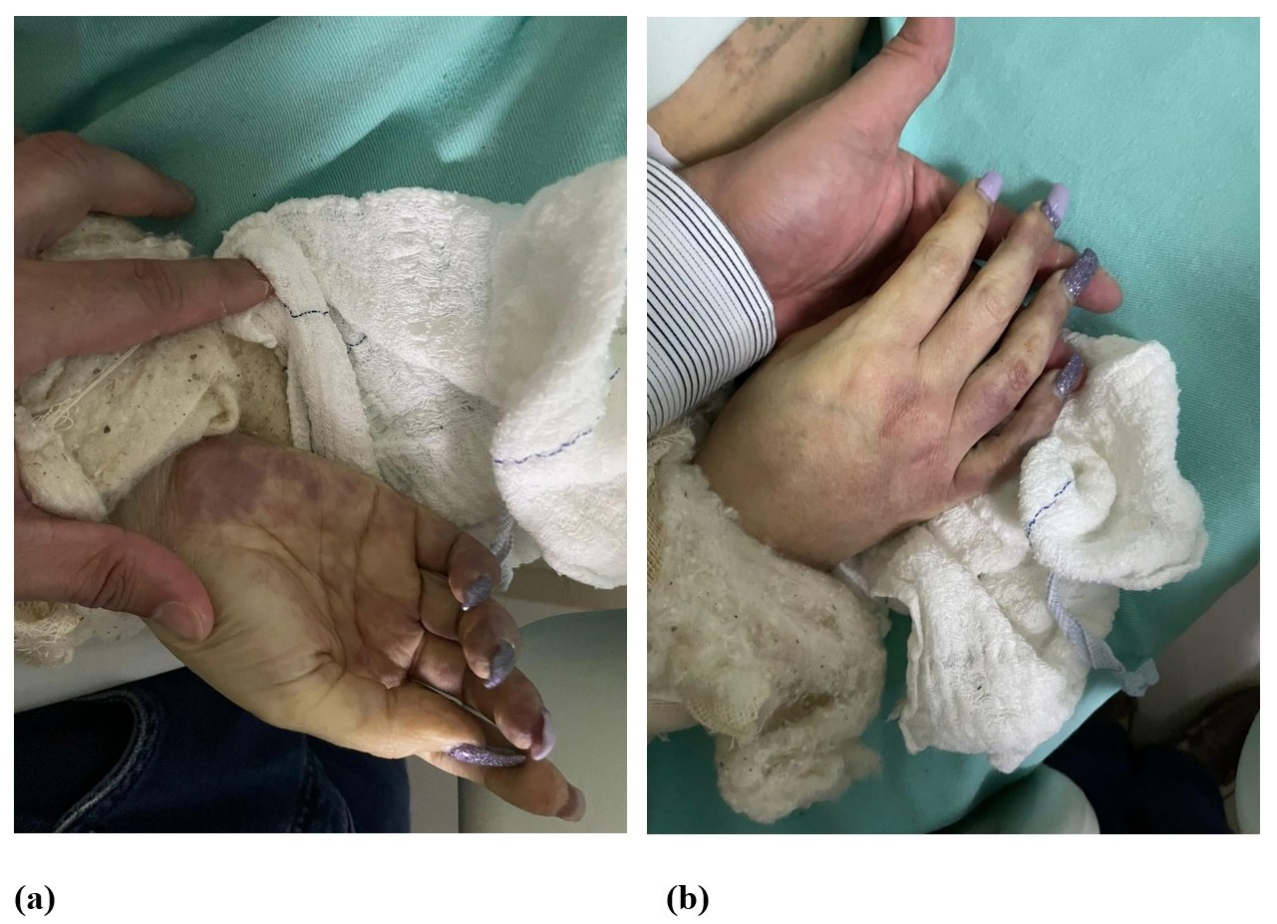
(a) and (b) Appearance of the hand after arterial occlusion.

Due to difficulty obtaining peripheral venous access, the patient was transferred to the surgical center, where venous access was attempted by puncture of the right wrist, in an area close to the anatomical snuffbox. A short time after the infusion was started, she complained of intense pain, accompanied by paresthesia, dormancy, coldness, and cyanosis of the hand and fingers (Figures 1a and 1b). The brachial, radial, and ulnar pulses were absent on physical examination.

**Figure 2:**
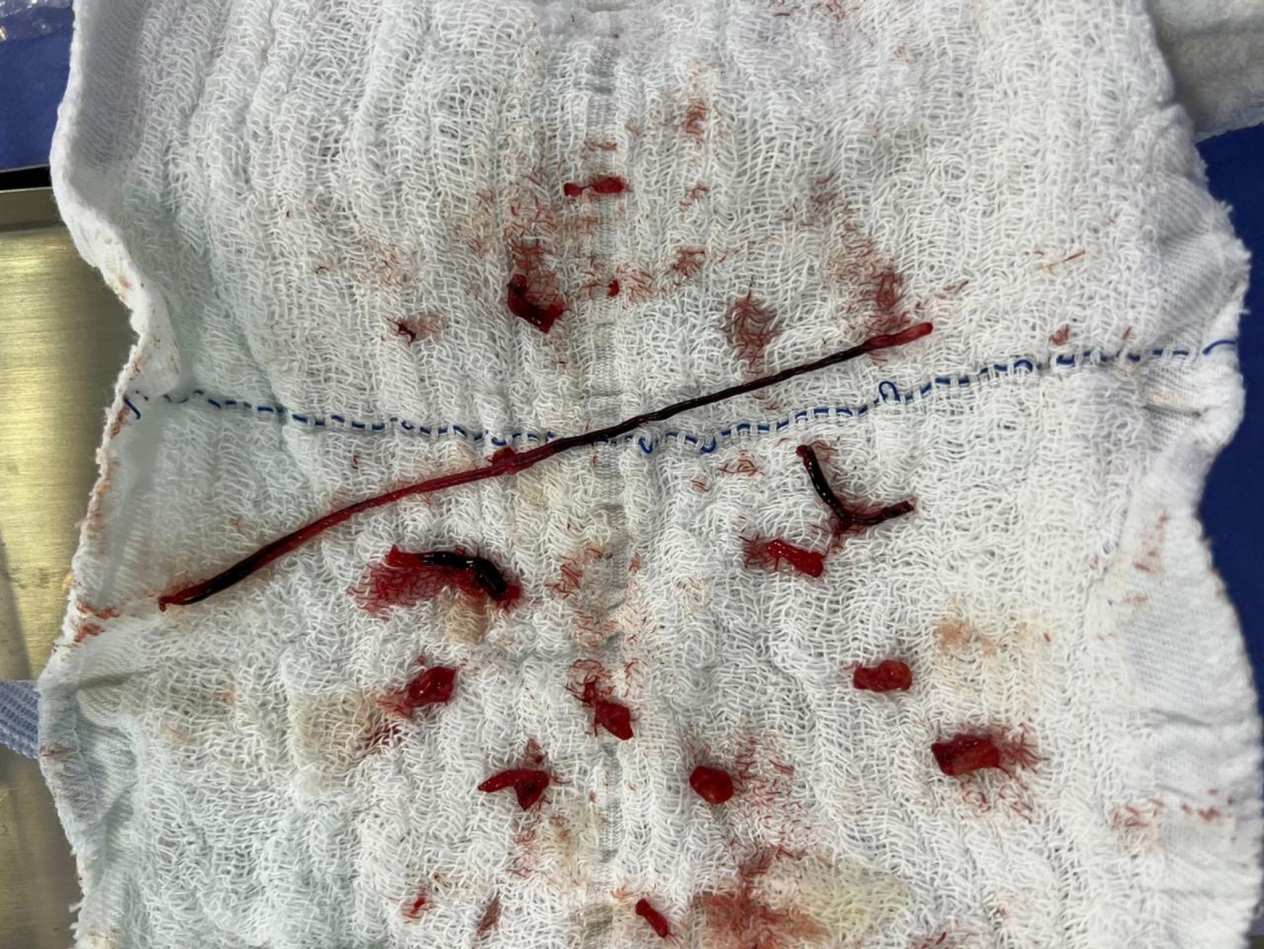
Fragments of thrombi after embolectomy.

The patient was promptly examined by a vascular surgeon, who conducted a Doppler ultrasonography examination of the arteries of the right upper limb, identifying occlusion of the brachial, radial, and ulnar arteries and observing thrombi with a recent appearance along the entire length of these arteries. She was taken to the surgical center, where she underwent arterial embolectomy via a brachial artery access in the cubital fossa, using Fogarty catheters numbers 3 and 4. The procedure removed large quantities of thrombi with acute characteristics (Figure 2). At the end of this intervention, the brachial, radial, and ulnar pulses were observed to be present and there was adequate perfusion of the right hand. The patient was kept on full anticoagulation (enoxaparin sodium 1 mg/kg every 12 h).

**Figure 3:**
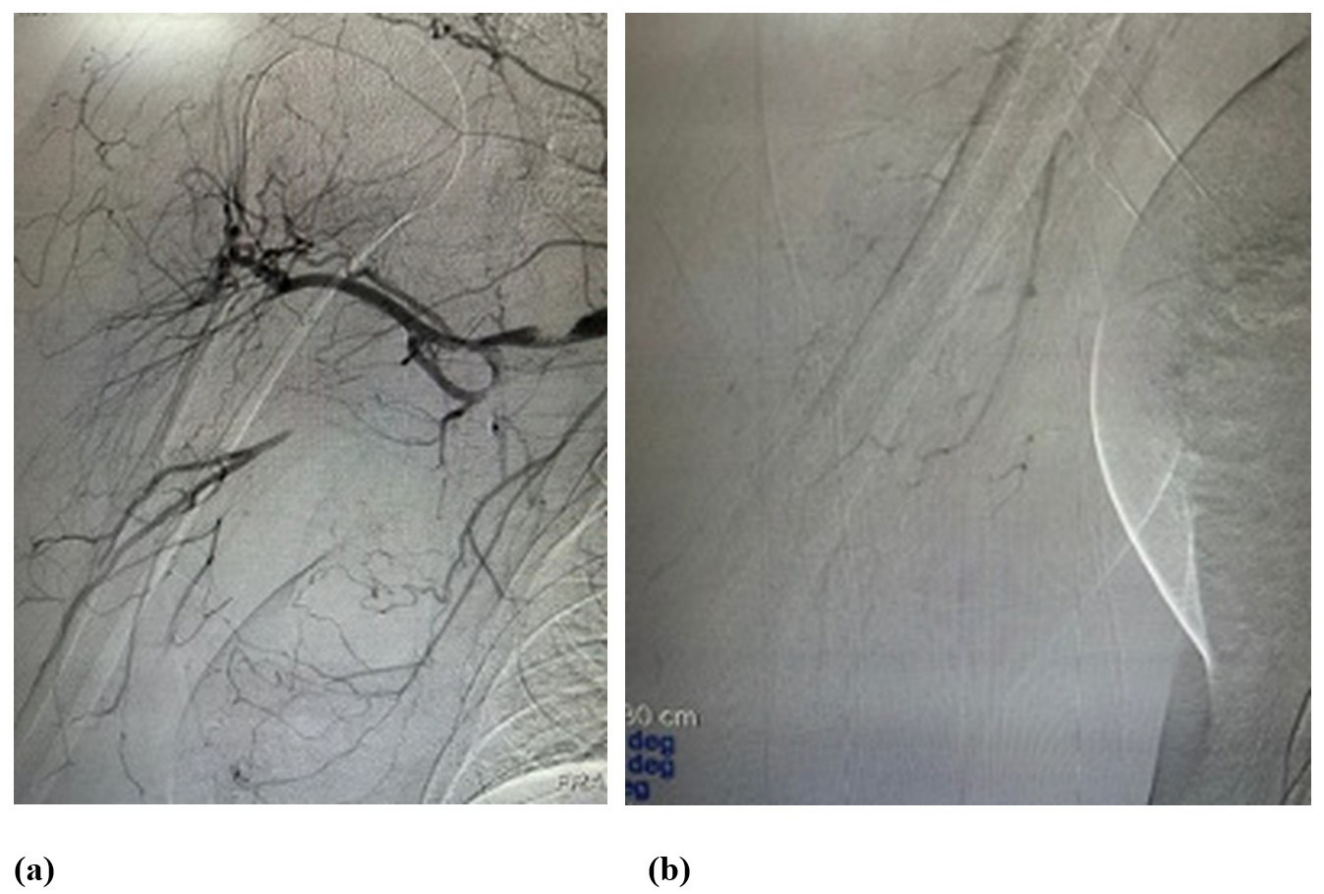
(a) and (b) Preoperative angiography.

Some hours after the procedure, the patient’s clinical status relapsed, and arterial embolectomy was performed again using the same devices as previously. The procedure was again successful and the patient was kept on full anticoagulation (enoxaparin sodium 1 mg/kg every 12 h). On the following day, the patient once more exhibited signs compatible with acute arterial occlusion of the right upper limb. This time she was taken to the catheterization laboratory, where she underwent arteriography, which demonstrated occlusion of the axillary artery and opacification of the distal vessels of the right upper limb (Figures 3a and 3b).

**Figure 4:**
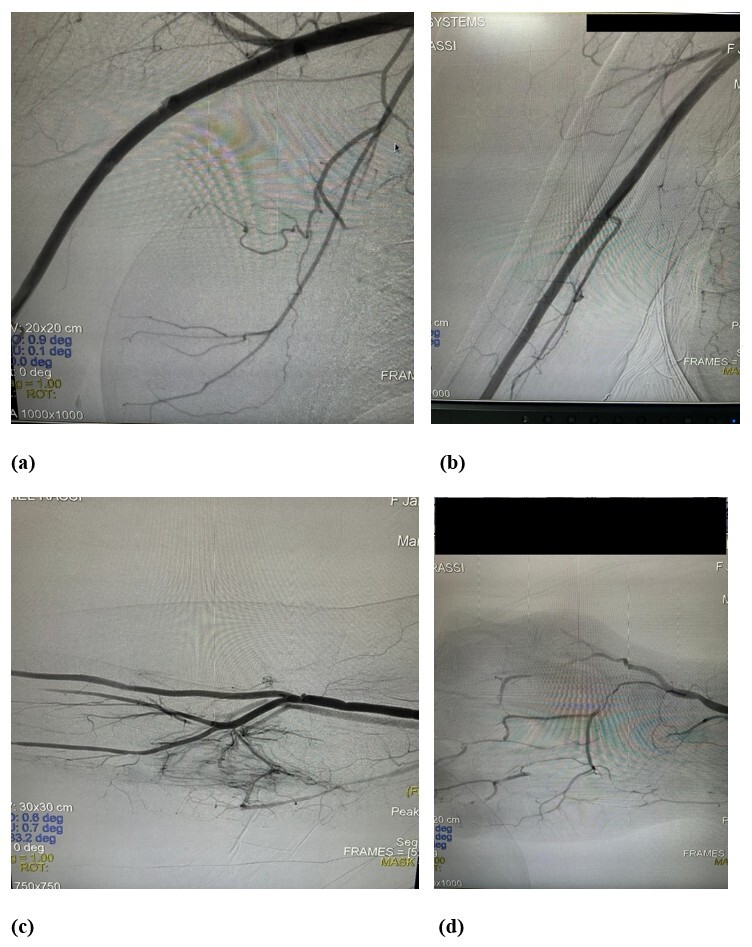
(a-d) Postoperative angiography.

In view of this clinical status, it was decided to perform embolectomy of the brachial, radial, and ulnar arteries, with access obtained via the cubital fossa and the distal third of the right forearm. Additionally, a 10 mg dose of a thrombolytic agent (alteplase) was administered directly into the arteries of the palmar arches, by intra-arterial infusion with a number 4 Nelaton probe, which was successful. Control arteriography demonstrated the vessels were patent and physical examination found the radial pulse was present and digital perfusion was good, with adequate oximetry readings (Figures 4a-d).

However, a few hours later, the patient suffered another episode of acute arterial occlusion. From this point on, clinical treatment was continued with full parenteral anticoagulation (enoxaparin sodium 1 mg/kg every 12 h) and administration of prostaglandin E1 (Alprostadil alfadex® 80 mcg every 12 h).

**Figure 5:**
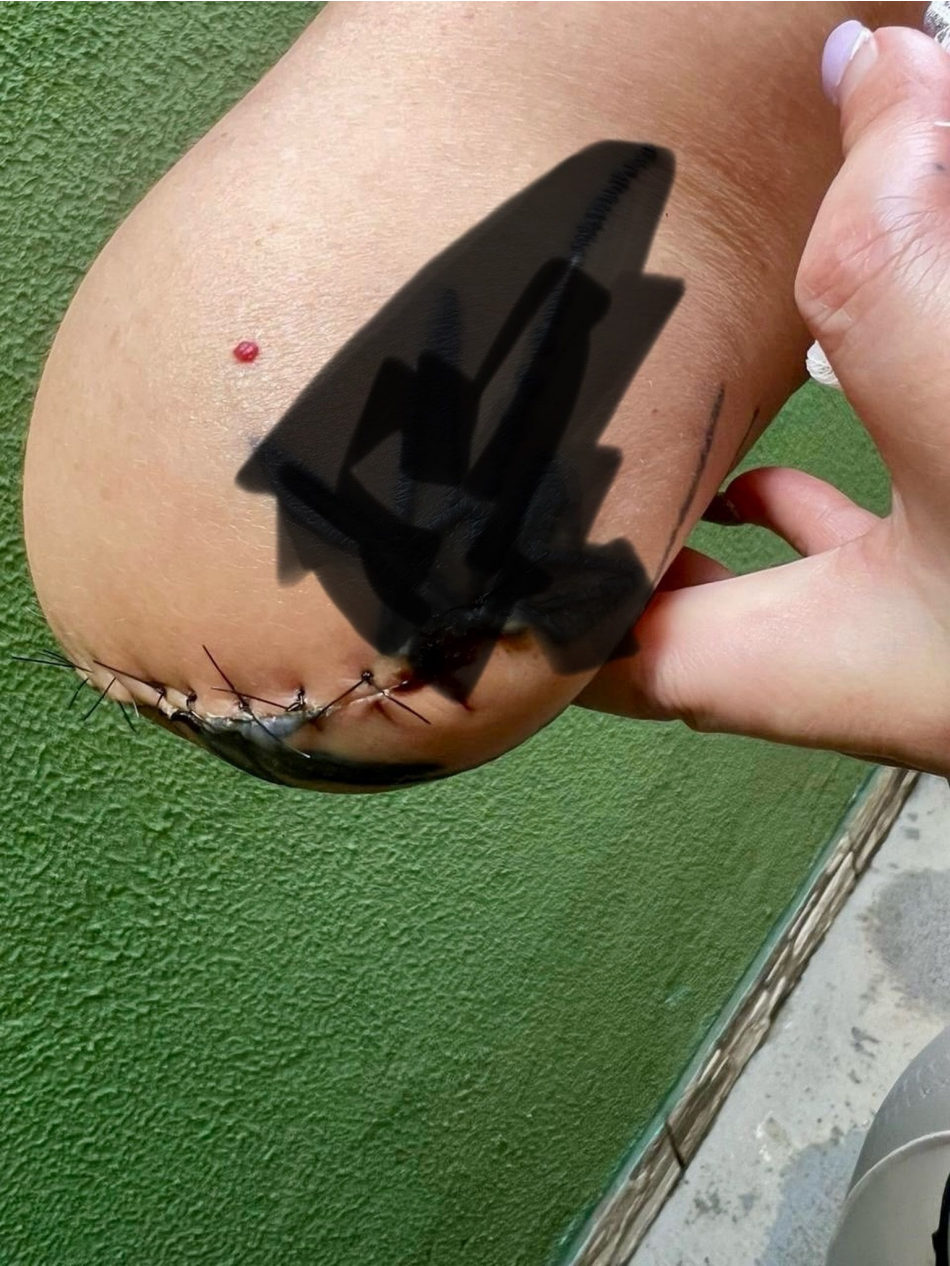
Amputation stump.

Despite these measures, the patient’s right upper limb developed gangrene and required surgical amputation just above the cubital fossa (Figure 5). The amputation stump healed well and there were no further complications. The sequence of clinical events is described in Table 1.

**Table 1:** Timeline of the clinical case.

**Date/Period**	**Event**
Day 0	Infusion of two units of packed red blood cells after plastic surgery.
Day 1	Prescription of intravenous Noripurum® with inadvertent infusion into the radial artery.
Day 1 (after infusion)	Intense pain, paresthesia and cyanosis of right upper limb; diagnosis of acute arterial occlusion.
Day 1 (after first embolectomy)	First arterial embolectomy (Fogarty catheters sizes 3 and 4) with removal of acute thrombi.
Day 1 (after second embolectomy)	De novo arterial occlusion; second embolectomy, initially successful.
Day 2	Arteriography shows occlusion of the axillary artery; third embolectomy + thrombolysis.
Following days	Recurrent arterial occlusion; clinical management with full anticoagulation and prostaglandins.
Day 7	Progression to gangrene and amputation above the cubital fossa.

**Figure 6:**
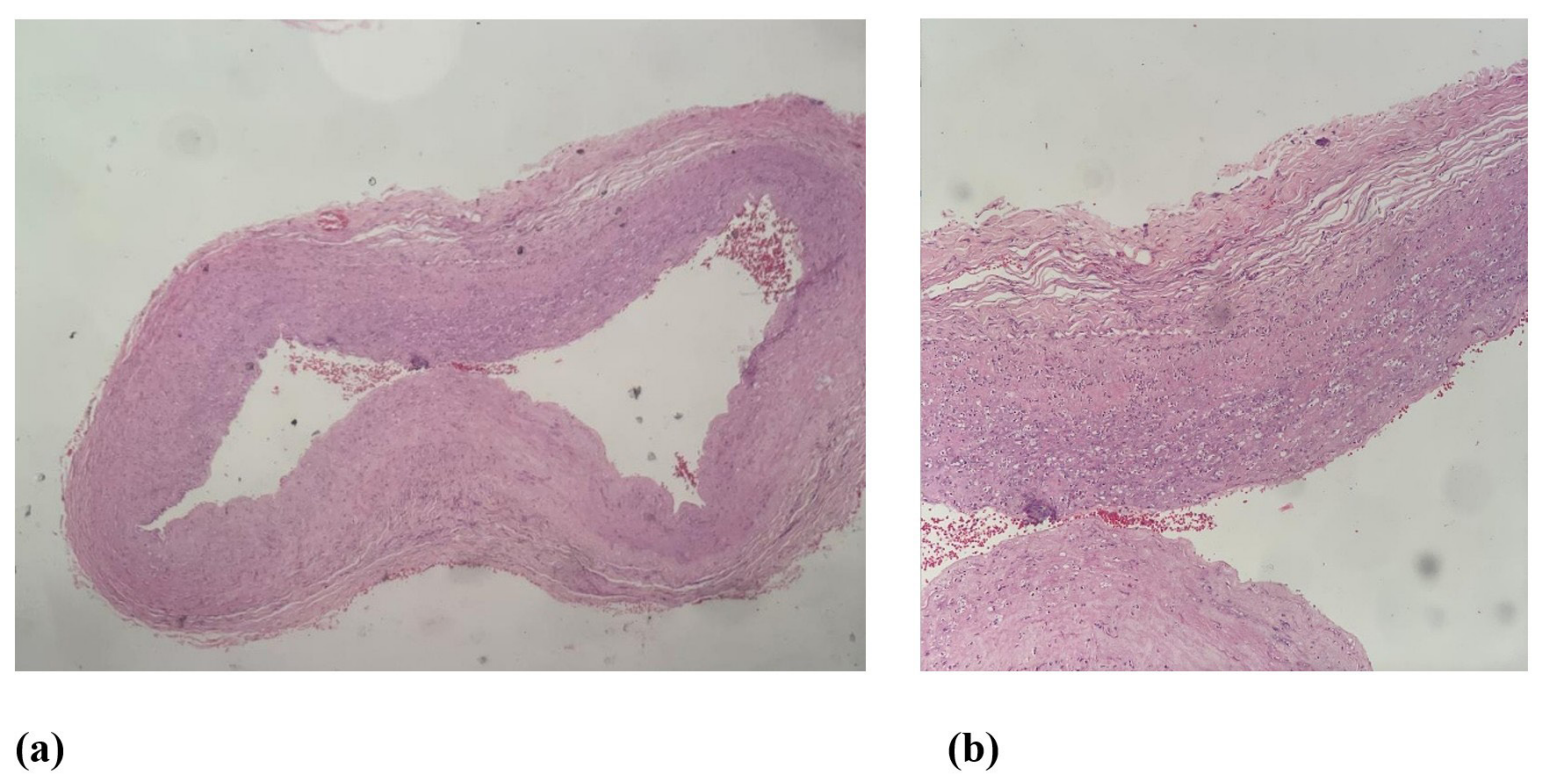
(a) and (b) Histopathological slides.

Specimens of proximal and distal arterial segments were sent for anatomic pathology analysis, which showed foci of endothelial destruction and focal areas of vascular wall necrosis, associated with transmural neutrophil infiltration, compatible with neutrophilic vasculitis (Figures 6a and 6b).

## DISCUSSION

Inadvertent intra-arterial infusion of substances or medications is well-documented in the scientific literature, which describes manifestations along a varied clinical spectrum, ranging from local cutaneous lesions to serious complications, such as loss of limbs.^12,13^

The first study to demonstrate direct evidence that iron overload provokes ROS production in the vascular wall was conducted by Greenwood et al.^14^ These authors observed that accumulation of iron increased lipid peroxidation and local production of ROS, corroborating the hypothesis that excess iron amplifies oxidative stress *in vivo*. In animal models, particularly with rats, it has also been observed that vascular relaxation is impaired, attributed to reduced NO bioavailability, contributing further evidence of the link between oxidative stress and endothelial dysfunction.^6^

In another study, Rooyakkers et al.^15^ demonstrated that acute infusion of ferric saccharate at dosages commonly used for intravenous supplementation in patients on hemodialysis resulted in an up to four times increase in levels of non-transferrin-bound iron, in addition to provoking a transitory reduction in vascular wall relaxation. Non-transferrin-bound iron can act as a catalyst of ROS formation, contributing to endothelial toxicity. The study concluded that administration of ferric saccharate at therapeutic doses can cause reversible endothelial dysfunction in healthy individuals.

In the present case, pathology results for the arterial specimen revealed a picture compatible with neutrophilic vasculitis, probably triggered by the agent that had been infused. Known pathophysiologic mechanisms underlying this type of vasculitis include an aberrant inflammatory response, often associated with paraneoplastic or drug-induced conditions.

From a surgical point of view, this case illustrates the difficulty of managing severe and recurrent acute arterial occlusion. Despite immediate and successive embolectomy procedures, which restored perfusion, each procedure was followed by early relapse of thrombosis, resulting in multiple interventions over a short period of time. Addition of intra-arterial thrombolysis to surgical treatment was also insufficient to prevent progression of the ischemic condition, culminating in major amputation of the involved limb. This outcome underscores the importance of early surgical intervention, but also demonstrates its limitations in scenarios of extensive endothelial injury, in which even repeated interventions may not ensure limb viability.

### Patient consent

The patient gave her consent to publication of this case report, including the images and clinical data presented.

### Ethical considerations

This study was submitted for Research Ethics Committee analysis, via the Plataforma Brasil system, and was approved with Substantiated Opinion Number 7.472.451 and Ethics Appraisal Submission Certificate 85927624.3.0000.0035.

## CONCLUSIONS

Analysis of the present case report supports the inference that intra-arterial iron administration can constitute a significant risk factor for occurrence of ischemic arterial events, secondary to endothelial injury and thrombosis. This risk appears to be linked to changes affecting both the coagulation cascade and the integrity and functionality of the endothelium.

## Data Availability

Os dados que sustentam este estudo estão disponíveis mediante solicitação ao autor correspondente, DCMF, por se tratar de dados relativos a um paciente, garantindo assim a sua privacidade.
