# Supplementary material for: Acute arterial occlusion following inadvertent infusion of ferric hydroxide sacarate (Noripurum®) into the radial artery: case report
Source: J Vasc Bras. 2026 Mar 23;25:e20250055. doi: 10.1590/1677-5449.202500552 (PMC13020970; doi:10.1590/1677-5449.202500552)
Supplement: Translation

# Translation: Oclusão arterial aguda após infusão inadvertida de sacarato de hidróxido férrico (Noripurum®) na artéria radial: relato de caso

**DOI:** 10.1590/1677-5449.202500551

**Authors:** Daniel César Magalhães Fernandes, Daniel Rassi Gusmao, Felipe Mendonça Oliveira e Souza, Juliano Ricardo Santana dos Santos, Beatriz Franco Fidalgo

**Affiliations:** 1 Hospital Geral de Goiânia – HGG, Goiânia, GO, Brasil.; 2 Instituto de Angiologia de Goiânia – IAG, Goiânia, GO, Brasil.; 3 Universidade Federal de Goiás – UFG, Hospital das Clínicas, Goiânia, GO, Brasil.; 4 Einstein, Hospital Municipal de Aparecida – HMAP, Goiânia, GO, Brasil.

**Keywords:** oclusão arterial, estresse oxidativo, vasculite, relato de caso

## Abstract

A sobrecarga de ferro, seja aguda ou crônica, tem sido associada a eventos adversos na patogênese de doenças cardiovasculares isquêmicas. No entanto, os efeitos do excesso de ferro na função vascular periférica e na resposta trombótica à lesão endotelial ainda não são bem compreendidos. Reportamos um caso de infusão arterial inadvertida de sacarato de hidróxido férrico (Noripurum®), que ocasionou um quadro de oclusão arterial aguda severa por provável lesão endotelial difusa e que, mesmo após tratamento cirúrgico e terapia medicamentosa, não evoluiu satisfatoriamente. Como não encontramos relatos semelhantes na literatura nas principais bases de dados disponíveis e por envolver reações complexas ainda não compreendidas entre sobrecarga de ferro e lesão endotelial dos vasos, optamos por fazer este relato.

## INTRODUÇÃO

Embora o ferro seja essencial para diversos processos fisiológicos, seu excesso pode desencadear lesões teciduais por meio da produção de radicais livres de oxigênio (RLOs), originados a partir de reações complexas^1^ . A sobrecarga secundária de ferro pode ser resultado de transfusões sanguíneas frequentes ou infusões parenterais da substância^2^ . Apesar de controvérsias existentes, diversas linhas de evidência sugerem que o excesso de ferro pode predispor ao desenvolvimento de doenças vasculares. Níveis elevados de reservas corporais de ferro foram associados a um risco aumentado de infarto do miocárdio em uma grande coorte de homens na Finlândia^3^ .

Estudos *in vitro* demonstraram que o ferro induz de forma aguda a agregação plaquetária^4^ , ao passo que a quelação do ferro pode inibir a expressão do fator tecidual^5^ . No entanto, os efeitos da sobrecarga crônica e aguda de ferro sobre o endotélio e a cascata de coagulação ainda permanecem pouco compreendidos.

O ferro também pode exercer efeitos diretos sobre a estrutura vascular, uma vez que a geração local de RLOs reduz a biodisponibilidade de óxido nítrico (ON), comprometendo o relaxamento da parede vascular e favorecendo, por consequência, a adesão e a agregação plaquetária^6^ . Dessa forma, observa-se uma relação entre disfunção endotelial e trombose, ambos potencialmente mediados pelo estresse oxidativo^7^ .

Estudos recentes demonstraram que, após infusões intravenosas de ferro para suplementação, ocorre um aumento superior a cinco vezes nos níveis de ferro não ligado à transferrina. Embora o significado clínico desse aumento ainda não esteja plenamente elucidado, evidências *in vitro* indicam que essa forma de ferro pode atuar como catalisador na formação de RLOs tóxicos^8^ . Esses mesmos estudos ressaltam a importância do endotélio — em especial, do ON endotelial — como um mecanismo de defesa primário contra o desenvolvimento de lesões ateroscleróticas^9,10^ .

Asperti and Vinchi^11^ , em modelo murino com sobrecarga aguda de ferro associada a reação hemolítica, demonstraram lesão endotelial mediada por ferro e estresse oxidativo, revertida parcialmente com o uso de *carbon monoxide-releasing molecules*, um liberador de monóxido de carbono terapêutico^11^ .

Diante das alterações induzidas pelo ferro sobre o endotélio vascular, e com o objetivo de aprofundar a compreensão dessa interação e suas possíveis complicações, apresentamos, a seguir, o relato de caso descrito neste artigo.

## RELATO DE CASO

Paciente do sexo feminino, 41 anos de idade, em pós-operatório recente de cirurgia plástica, procurou o pronto-socorro apresentando quadro de fraqueza, sem outras queixas associadas. Na ocasião, foram administradas duas unidades de concentrado de hemácias. No dia seguinte, a paciente retornou ao pronto atendimento com sintomas semelhantes, sendo então prescrita infusão endovenosa de sacarato de hidróxido férrico (Noripurum®).

Devido à dificuldade de obtenção de acesso venoso periférico, a paciente foi encaminhada ao centro cirúrgico, onde foi puncionado um acesso venoso no punho direito, em topografia próxima à tabaqueira anatômica. Pouco tempo após o início da infusão, relatou dor de forte intensidade, acompanhada de parestesia, dormência, frialdade e cianose da mão e dos dedos (Figuras 1a e 1b), além de ausência de pulsos braquial, radial e ulnar ao exame físico.

**Figura 1:** (a) e (b) Aspecto da mão após oclusão arterial.

A paciente foi prontamente avaliada por cirurgião vascular, que realizou eco-Doppler arterial do membro superior direito, identificando oclusão das artérias braquial, radial e ulnar, com visualização de trombos de aspecto recente ao longo de todo o trajeto arterial. Em seguida, foi encaminhada ao centro cirúrgico, onde foi submetida à embolectomia arterial por meio de acesso na fossa cubital à artéria braquial, utilizando-se cateteres de Fogarty números 3 e 4. O procedimento resultou na exteriorização de grande quantidade de trombos com características agudas (Figura 2). Ao término da intervenção, observou-se presença de pulsos braquial, radial e ulnar, bem como perfusão adequada da mão direita, tendo sido mantida a paciente em anticoagulação plena (enoxaparina sódica 1 mg/kg de 12/12 h).

**Figura 2:** Fragmentos de trombos após embolectomia.

Algumas horas após o procedimento, houve recidiva do quadro clínico, sendo indicada nova embolectomia arterial com a utilização dos mesmos dispositivos empregados anteriormente, procedimento que também foi realizado com sucesso, e novamente mantida anticoagulação plena (enoxaparina sódica 1 mg/kg de 12/12 h). No dia seguinte, a paciente voltou a apresentar sinais compatíveis com oclusão arterial aguda do membro superior direito. Dessa vez, foi encaminhada ao setor de hemodinâmica, onde foi submetida à arteriografia, que demonstrou oclusão da artéria axilar, com ausência de opacificação dos vasos distais do membro superior direito (Figuras 3a e 3b).

**Figura 3:** (a) e (b) Angiografia pré-operatória.

Diante desse quadro clínico, optou-se por uma embolectomia das artérias braquial, radial e ulnar, com acessos pela fossa cubital e pelo terço distal do antebraço direito. Adicionalmente, foi administrado trombolítico (alteplase) diretamente nas artérias do arco palmar, por meio de infusão intra-arterial com sonda de Nelaton número 04, na dose de 10 mg, com sucesso. A arteriografia de controle demonstrou vasos pérvios, e o exame físico evidenciou pulso radial presente e boa perfusão digital, com oximetria adequada (Figuras 4a-d).

**Figura 4:** (a-d) Angiografia pós-operatória.

Entretanto, poucas horas depois, a paciente apresentou novo episódio de oclusão arterial aguda. A partir desse momento, optou-se por tratamento clínico com anticoagulação plena por via parenteral (enoxaparina sódica 1 mg/kg de 12/12 h) e administração de prostaglandina E1 (Alprostadil alfadex® 80 mcg de 12/12 h).

Apesar das medidas instituídas, a paciente evoluiu com gangrena do membro superior direito, sendo necessária amputação cirúrgica logo acima da fossa cubital (Figura 5), com o coto de amputação evoluindo com boa cicatrização e sem outras complicações. A linha temporal dos eventos clínicos está resumida na Tabela 1.

**Figura 5:** Coto amputação.

**Tabela 1:** Linha do tempo do caso clínico.

**Data/Período**	**Evento**
Dia 0	Infusão de duas bolsas de hemácias após cirurgia plástica.
Dia 1	Prescrição de Noripurum® endovenoso e infusão inadvertida em artéria radial.
Dia 1 (após infusão)	Dor intensa, parestesia e cianose em membro superior direito; diagnóstico de oclusão arterial aguda.
Dia 1 (pós-primeira embolectomia)	Primeira embolectomia arterial (uso de cateter de Fogarty 3 e 4) com retirada de trombos agudos.
Dia 1 (pós-segunda embolectomia)	Nova oclusão arterial; segunda embolectomia com sucesso inicial.
Dia 2	Arteriografia mostra oclusão em artéria axilar; terceira embolectomia + trombólise.
Dias seguintes	Reoclusão arterial recorrente; manejo clínico com anticoagulação plena e prostaglandinas.
Dia 7	Evolução para gangrena e amputação acima da fossa cubital.

Foram enviados segmentos arteriais proximais e distais para análise anatomopatológica, que evidenciou focos de destruição endotelial e áreas de necrose focal da parede vascular, associadas a infiltrado neutrofílico transmural, compatíveis com vasculite neutrofílica (Figuras 6a e 6b).

**Figura 6:** (a) e (b) Lâminas do histopatológico.

## DISCUSSÃO

A infusão inadvertida de substâncias ou medicamentos por via intra-arterial, com consequente manifestação de um espectro clínico variado — desde lesões cutâneas locais até complicações graves, como perda de membros —, está bem documentada na literatura científica^12,13^ .

O primeiro estudo a demonstrar evidência direta de que a sobrecarga de ferro promove a geração de RLOs na parede vascular foi conduzido por Greenwood et al.^14^ . Os autores observaram que o acúmulo de ferro aumentava a peroxidação lipídica e a produção local de espécies reativas de oxigênio, corroborando a hipótese de que o excesso de ferro amplifica o estresse oxidativo *in vivo*. Em modelos animais, particularmente em ratos, também se observou prejuízo no relaxamento vascular, atribuído à redução da biodisponibilidade de ON, reforçando a ligação entre estresse oxidativo e disfunção endotelial^6^ .

Em outro estudo, Rooyakkers et al.^15^ demonstraram que a infusão aguda de sacarato férrico, em doses comumente utilizadas para suplementação endovenosa em pacientes em hemodiálise, induz um aumento de até quatro vezes nos níveis de ferro não ligado à transferrina, além de provocar uma redução transitória no relaxamento da parede vascular. O ferro não ligado à transferrina pode agir como catalisador na formação de RLOs, contribuindo para a toxicidade endotelial. O estudo concluiu que a administração de sacarato férrico em dose terapêutica pode causar disfunção endotelial reversível em indivíduos saudáveis.

No presente relato, o exame anatomopatológico de segmento arterial demonstrou um quadro compatível com vasculite neutrofílica, provavelmente desencadeada pelo agente infundido. Entre os mecanismos fisiopatológicos reconhecidos para esse tipo de vasculite, destaca-se a resposta inflamatória aberrante, frequentemente associada a quadros paraneoplásicos ou induzidos por fármacos.

Do ponto de vista cirúrgico, este caso ilustra a dificuldade de manejo de uma oclusão arterial aguda grave e recorrente. Apesar da realização imediata de embolectomias sucessivas, com restauração temporária da perfusão, observou-se recidiva precoce da trombose, exigindo múltiplas intervenções em um curto intervalo de tempo. A associação de trombólise intra-arterial ao tratamento cirúrgico também não foi suficiente para evitar a progressão do quadro isquêmico, que culminou em amputação maior do membro acometido. Esse desfecho reforça a importância da abordagem cirúrgica precoce, mas também evidencia suas limitações em cenários de lesão endotelial extensa, nos quais mesmo intervenções repetidas podem não garantir a viabilidade do membro.

### Nota de consentimento

A paciente consentiu com a publicação deste relato de caso, incluindo as imagens e os dados clínicos apresentados.

### Aspectos éticos

O presente trabalho foi submetido à avaliação do Comitê de Ética em Pesquisa, por meio da Plataforma Brasil, sendo aprovado sob o número de parecer consubstanciado 7.472.451 e Certificado de Apresentação de Apreciação Ética 85927624.3.0000.0035.

## CONCLUSÃO

A partir da análise do presente relato de caso, é possível inferir que a administração de ferro intra-arterial pode representar um fator de risco significativo para a ocorrência de eventos isquêmicos arteriais, secundários à lesão endotelial e trombose. Tal risco parece estar relacionado a alterações tanto na cascata de coagulação quanto na integridade e funcionalidade do endotélio.

## Data Availability

The data supporting this study are available upon request from the corresponding author, DCMF, as they pertain to a patient, thus ensuring their privacy.
